# Rapid Formation of Nanoclusters for Detection of Drugs in Urine Using Surface-Enhanced Raman Spectroscopy

**DOI:** 10.3390/nano11071789

**Published:** 2021-07-09

**Authors:** Yun-Chu Chen, Shang-Wen Hong, Huang-Hesin Wu, Yuh-Lin Wang, Yih-Fan Chen

**Affiliations:** 1 Institute of Biophotonics, National Yang Ming Chiao Tung University, Taipei 112, Taiwan; yunchu00@gmail.com (Y.-C.C.); joy0970515997@gmail.com (S.-W.H.); leowu0719@gmail.com (H.-H.W.); 2 Institute of Atomic and Molecular Sciences, Academia Sinica, Taipei 106, Taiwan; ylwang@pub.iams.sinica.edu.tw

**Keywords:** surface-enhanced Raman spectroscopy (SERS), drug, new psychoactive substance, urine, nanocluster

## Abstract

We developed a method based on surface-enhanced Raman spectroscopy (SERS) and a sample pretreatment process for rapid, sensitive, reproducible, multiplexed, and low-cost detection of illegal drugs in urine. The abuse of new psychoactive substances (NPS) has become an increasingly serious problem in many countries. However, immunoassay-based screening kits for NPS are usually not available because of the lack of corresponding antibodies. SERS has a great potential for rapid detection of NPS because it can simultaneously detect multiple kinds of drugs without the use of antibodies. To achieve highly sensitive SERS detection of drugs, sodium bromide was first employed to induce the rapid formation of Ag nanoclusters by aggregating silver nanoparticles (AgNPs) in the extracted sample solution. SERS measurements were performed immediately after the sample pretreatment without incubation. The three-dimensional SERS hot spots were believed to form significantly within the nanoclusters, providing strong SERS enhancement effects. The displacement of citrate molecules on the surfaces of the AgNPs by bromide ions helped increase the adsorption of drug molecules, increasing their areal density. We demonstrated the simultaneous detection of two kinds of NPS, methcathinone and 4-methylmethcathinone, in urine at a concentration as low as 0.01 ppm.

## 1. Introduction

Drug abuse has become an increasingly serious problem in many countries. The use of either traditional illegal drugs such as cocaine and heroin or new psychoactive substances (NPS) such as methcathinone (MC) and 4-methylmethcathinone (4-MMC, mephedrone) poses a serious threat to health and public security. To detect the use of illegal drugs during the preceding 1–4 days, urine samples are usually collected and analyzed using GC-MS or LC-MS/MS [1,2,3]. These methods can provide sensitive and specific detection of drugs; however, they need to be performed by well-trained personnel in a laboratory and require delicate sample pretreatment processes and expensive reagents. Therefore, for the rapid, on-site detection of illegal drugs, lateral flow immunoassays are usually used instead [4,5]. These assays are low-cost and easy-to-use, but they usually suffer from a higher false-positive rate [4]. Rapid screening of NPS is especially difficult because immunoassay test kits are usually not available due to the lack of corresponding antibodies and aptamers. Therefore, to prevent the spread of NPS, the development of a rapid, simple, sensitive, and low-cost method that can detect NPS in urine without the need of antibodies or aptamers is urgent.

Surface-enhanced Raman spectroscopy (SERS) has been used to detect a variety of molecules [6,7,8,9,10] because SERS-based methods only need a small sample and take a few seconds to achieve the multiplexed detection of molecules with high sensitivity. Many studies have reported the SERS detection of drugs, including cannabinoid [11], cocaine [12,13,14,15,16,17], flunitrazepam [18], alpha-methyltryptamine [19], heroin [17,20], ketamine [21], morphine [13,22], benzodiazepine [23], methamphetamine [13,24,25,26,27,28,29,30], amphetamine [31], opioid [32], 3,4-methylenedioxymethamphetamine (MDMA) [25,27,28,33], 4-MMC [34,35], MC [25], barbiturate [36], and sulfa drugs [37]. While the plasmonic properties of nanostructured substrates or nanoparticles used for SERS measurements is critical for the sensitive detection of molecules, it has been demonstrated that sample pretreatment is also very important for the SERS detection of drugs in biological samples such as urine [14,25,27,29,30,36] and serum [30]. It should be noted that, although antibodies or aptamers are sometimes used in SERS-based detection methods [12], they are not required in all SERS detection methods, which makes it useful for detecting NPS.

Three-dimensional (3D) SERS substrates have drawn much attention in recent years [38]. Given that the laser focal volume is a 3D space, SERS substrates with SERS hot spots in all spatial directions can utilize the laser focal volume better than a 2D SERS substrate. In addition, having SERS hot spots in the z-axis allows for a higher tolerance for the position of the laser focal plane. Recent studies showed that 3D SERS substrates could be fabricated using nano-fabrication processes [39] or formed by metal colloidal nanoparticles [19,27,40,41,42,43]. For example, several studies demonstrated that a 3D SERS hot spot matrix could be formed by evaporating a droplet of metal nanoparticles to provide highly sensitive detection of molecules [19,40,41,42,43]. As the interparticle distance, which was critical to the SERS effect, gradually decreased during the evaporation process in those studies, the interparticle distance could decrease to an optimal value at a certain time point and resulted in strong SERS effects. On the other hand, instead of using the evaporation process, inducing the aggregation of nanoparticles was another strategy to generate 3D SERS hot spots [32,44]. The sensitive and quantitative SERS detection of molecules was demonstrated by adding a suitable kind and concentration of aggregating agent, such as an alkali halide salt [32,45], to colloidal metal nanoparticles to form nanoclusters with many SERS-active nanogaps.

In this study, we developed a sensitive and reproducible SERS method for the detection of drugs in urine. A simple sample pretreatment process was developed to reduce interferences from other components in urine on the detection results. An SERS measurement was performed immediately after sodium bromide (NaBr), a kind of alkali halide salt, was added to a mixture of the pretreated sample and a silver nanoparticle (AgNP) colloidal solution. By using an alkali halide salt as an aggregating agent to induce the rapid formation of Ag nanoclusters with SERS hot spots in three dimensions, the sensitive and reproducible detection of drugs was achieved without incubation time and a nanofabricated SERS substrate. We demonstrated individual and simultaneous detection of two kinds of NPS, MC and 4-MMC, in urine at a concentration as low as 0.01 ppm.

## 2. Materials and Methods

### 2.1. Materials

S(-)-methcathinone hydrochloride solution, 4-methylmethcathinone (mephedrone) hydrochloride solution, n-hexane, sodium chloride (NaCl), potassium bromide (KBr), and sodium borohydride (NaBH_4_) were purchased from Sigma-Aldrich. Sodium bromide (NaBr) and silver nitrate (AgNO_3_) were purchased from J.T. Baker. Trisodium citrate dihydrate and sodium hydroxide (NaOH) were purchased from Showa Chemical Industry. Ultrapure water was produced by a Millipore water system.

### 2.2. Synthesis of AgNPs

The glassware used for the synthesis of AgNPs was cleaned with aqua regia and was then rinsed with ultrapure water before use. To synthesize 4-nanometer AgNP seeds, 47.5 mL of 0.2% trisodium citrate, 0.85 mL of 60 mM AgNO_3_, and 1 mL of 0.1% NaBH_4_ were mixed at 70 °C in a reflux setup for 60 min. Next, to increase the size of the AgNPs, 5 mL of the synthesized 4-nanometer AgNP seeds, 1 mL of 1% trisodium citrate, 37.5 mL of ultrapure water, and 0.85 mL of 60 mM AgNO_3_ were mixed and boiled in a reflux setup for 60 min. Then, 1 mL of 1% trisodium citrate and 0.85 mL of 60 mM AgNO_3_ were added to the boiled mixture, and the mixture was boiled for another 60 min. The addition of trisodium citrate and AgNO_3_ and the boiling process were repeated two more times. Finally, the mixture was cooled to room temperature, and the AgNPs were ready to use. Transmission electron microscopy (TEM) images showed that the diameter of the AgNPs was ~28 nm. (Supplementary material Figure S1a). The localized surface plasmon resonance (LSPR) wavelengths of the 4-nanometer AgNP seeds and the 28-nanometer AgNPs were 391.9 nm and 395.1 nm, respectively (Supplementary material Figure S1b).

### 2.3. Urine Sample Pretreatment

**Scheme 1:**
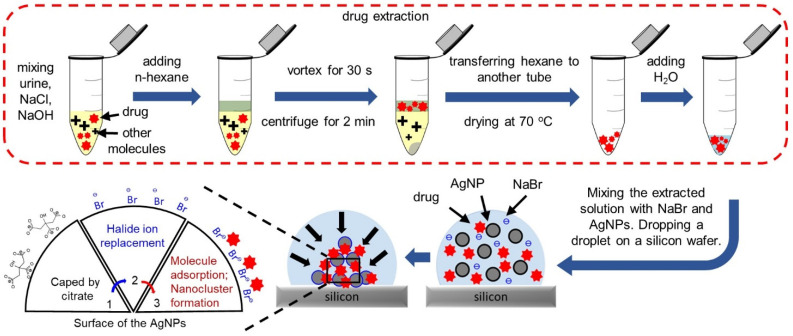
Illustration of the urine sample pretreatment and the formation of Ag nanoclusters for SERS detection of drugs in urine. A human urine sample is mixed with NaCl, NaOH, and n-hexane for drug extraction. NaBr was added to the extracted solution to induce rapid formation of Ag nanoclusters and to increase the adsorption of molecules on the AgNPs for SERS detection of drugs. A droplet of the mixture of AgNPs, NaBr, and the extracted sample solution was dropped onto a silicon wafer for SERS measurements.

The sample pretreatment process, which was modified from a process reported in the literature [25], is illustrated in Scheme 1. Briefly, human urine (1 mL) was mixed with 0.68 g of NaCl and 200 μL of 5% NaOH for 30 s. Then, n-hexane (200 μL) was added to the mixture, and the mixture was vigorously mixed by a vortex mixer for 30 s. After the mixing, the mixture was centrifuged at 9500 g for 2 min, and then the upper organic phase was transferred to another centrifuge tube. Finally, the collected n-hexane solution was dried at 70 °C, and ultrapure water (10 μL) was added to the centrifuge tube to resuspend the extracted drugs for SERS measurements.

### 2.4. SERS Measurement

To induce the rapid formation of Ag nanoclusters for SERS measurements, 0.5 µL of 250 mM NaBr was mixed with 5 µL of the synthesized 28 nm AgNPs and 5 µL of the sample solution (standard solution or extracted urine sample). The final concentration of NaBr in the mixture was ~12 mM. After the mixing, 5 µL of the mixture was dropped onto a silicon wafer for measurements. A 532-nanometer-Raman system (B&W TEK, i-Raman-532S) was used to measure the SERS spectra. The laser from the Raman probe was directed into a side port of an upright microscope (Olympus, BX51) and was focused by a 20X objective lens (N.A. 0.7) on a droplet of the mixture during SERS measurements.

## 3. Results and Discussion

### 3.1. Formation of SERS-Active Nanoclusters

The sample pretreatment and measurement processes for the SERS detection of drugs in urine are shown in Scheme 1. To extract drugs from urine, hexane and a large amount of NaCl were added to the samples first. NaCl was used to reduce the solubility of the drugs, and hexane was used to extract the drugs from the urine. After shaking the mixture of urine and hexane vigorously for 30 s, the upper phase containing hexane and the extracted drugs was transferred to another microcentrifuge tube and was then dried by heating. Finally, a small amount of water was added to the microcentrifuge tube to obtain a concentrated, resuspend drug solution for measurement. The whole sample pretreatment process could be finished within 20 min.

**Figure 1:**
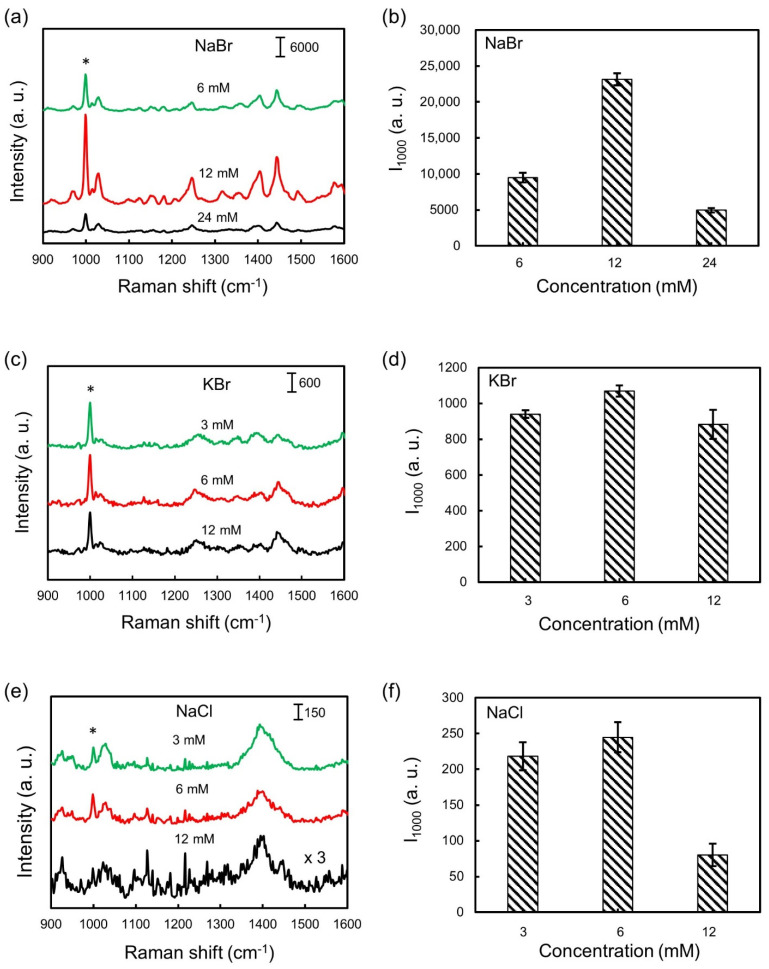
Effects of the kind and the concentration of alkali halide salt on SERS detection of drugs. (**a**,**c**,**e**) SERS spectra of 1 ppm MC standard solution measured using the nanoclusters induced by (**a**) NaBr, (**b**) KBr, and (**c**) NaCl of different concentrations. The characteristic Raman peak of MC at 1000 cm^−1^ is marked with “*”. (**b**,**d**,**f**) The intensities of the characteristic Raman peak of MC at 1000 cm^−1^ measured using the nanostructures induced by (**b**) NaBr, (**d**) KBr, and (**f**) NaCl of different concentrations. Error bars show standard deviations with n = 30.

To detect drugs using SERS, an alkali halide salt, which functioned as an aggregating agent, and AgNPs were added to a sample solution before the SERS measurements. It has been known that alkali halide ions can replace citrate molecules on the surfaces of AgNPs and reduce the stability of colloidal nanoparticles [32]. Depending on the kind and the concentration of the alkali halide salt mixed with the AgNPs, the AgNPs may form many small nanoclusters or large aggregates. However, for sensitive SERS detection of molecules, small nanoclusters are generally preferred over large aggregates because the molecules of interest can access the nanogaps among nanoparticles more easily. To find a suitable kind and concentration of alkali halide salt to induce the formation of nanoclusters for SERS detection, we measured 1 ppm MC using three kinds of alkali halide salts, NaCl, NaBr, and KBr, with different concentrations, as shown in Figure 1a,c,e. The range of the concentrations tested was different for the three kinds of alkali halide salts because they had different effects on the stability of the AgNPs. For each SERS measurement, we quickly mixed the drug solution, the 28-nanometer AgNPs, and one of the kinds of alkali halide salt, and then put a droplet of the mixture on a silicon wafer. The SERS spectra were measured immediately after the mixing without incubation. The droplet was not covered by a coverslip, and the 532-nanometer laser of the Raman system was focused on the droplet for measuring the SERS spectra. Figure 1b,d,f show that the intensity of the characteristic Raman peak of MC at 1000 cm^−1^ varied with the kind and the concentration of the alkali halide salt. Among the nine experimental conditions tested, we found that the intensity of the 1000 cm^−1^ peak was highest when 12 mM NaBr was mixed with the AgNPs and the sample solution. Therefore, we used this amount of NaBr to induce the formation of Ag nanoclusters for the other experiments of this study. 

The rapid formation of nanoclusters in a sample solution allowed us to detect molecules using SERS without incubation time. According to the extinction spectra of the AgNPs, the LSPR peak of the AgNP solution increased from 395.1 to 399.7 nm right after the addition of NaBr but then remained almost unchanged afterwards (Supplementary material Figure S1c,d). The dynamic light scattering (DLS) measurements also showed that the addition of NaBr caused an increase in the hydrodynamic diameter of the AgNPs (Supplementary material Figure S2a,b). Both the red shift in the LSPR peak and the increase in the hydrodynamic diameter indicated the formation of nanoclusters. A time-lapse observation of the SERS spectra of 1 ppm MC after the addition of 12 mM NaBr showed a slight increase in the Raman signal over time (Supplementary material Figure S3), which was likely caused by the evaporation-induced concentration of molecules. As the rate of evaporation depends on several factors, such as the humidity and temperature of the surrounding environment, to avoid the effect of evaporation on the detection, we performed the SERS measurements immediately after we put a droplet of the sample on a silicon wafer.

It should be noted that mixing the AgNPs with NaBr also helped to increase the adsorption of the drug molecules to the AgNPs because the citrate molecules capped on the surfaces of the AgNPs were displaced with bromide ions [32]. The Raman signals of the drug molecules adsorbed on the AgNPs could be significantly enhanced because of the SERS effects of the nanoclusters. On the other hand, the intensity of the characteristic Raman peak of citrate molecules at ~1400 cm^−1^ was not very strong in the measured spectra. It was most likely because the majority of the citrate molecules on the surfaces of the AgNPs were displaced by bromide ions during the displacement and aggregation processes that led to the formation of SERS hot spots.

### 3.2. Detection of MC and 4-MMC in Water

**Figure 2:**
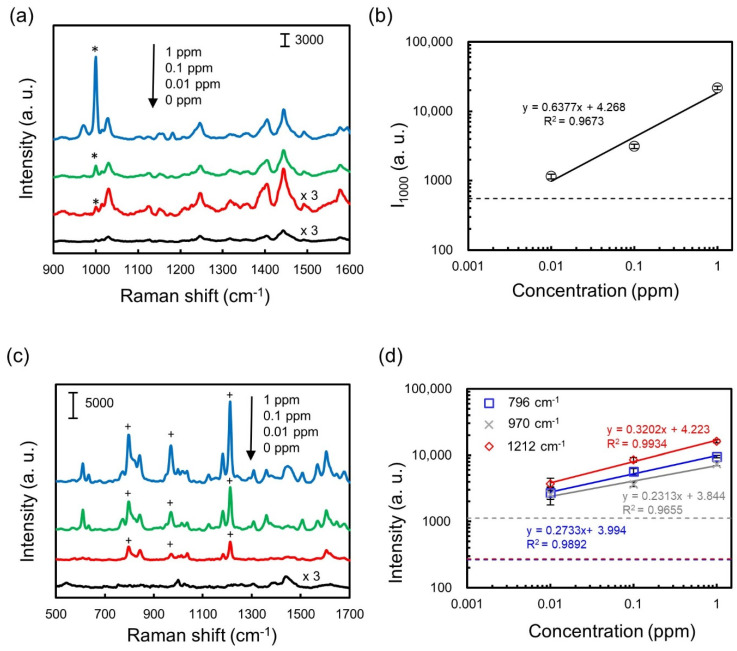
SERS detection of MC and 4-MMC in water. (**a,c**) SERS spectra of (**a**) MC and (**c**) 4-MMC in water with concentrations ranging from 0.01 to 1 ppm. The characteristic Raman peak of MC at 1000 cm^−1^ and the characteristic Raman peak of 4-MMC at 1212 cm^−1^ are marked with “*” and “+”, respectively. (**b**) A plot of the intensity of the 1000 cm^−1^ peak versus MC concentration. Error bars show standard deviations with n = 30. The straight line is the linear regression line, and the dotted line indicates mean plus 3 standard deviations (mean + 3σ, σ is the standard deviation) of the 1000 cm^−1^ peak of the blank sample. (**d**) A plot of the intensities of the 796, the 970, and the 1212 cm^−1^ peaks versus 4-MMC concentration. Error bars show standard deviations with n = 30. The straight lines are the linear regression lines, and the blue, gray, and red dotted lines indicate mean plus 3σ of the 796, the 970, and the 1212 cm^−1^ peaks, respectively, of the blank sample.

After finding the suitable kind and concentration of the aggregating agent, we used the optimal experimental condition to measure MC and 4-MMC standard solutions of different concentrations. Figure 2a shows the SERS spectra of MC with a concentration between 0.01 and 1 ppm, and Figure 2b shows the relationship between the concentration of MC and the intensity of the characteristic Raman peak at 1000 cm^−1^. Similarly, Figure 2c shows the SERS spectra of 4-MMC with a concentration between 0.01 and 1 ppm, and Figure 2d shows the relationships between the concentration of 4-MMC and the intensities of the three characteristic Raman peaks at 799, 970, 1212 cm^−1^, respectively. Our results showed that MC and 4-MMC could be detected within 1 min with high sensitivity using the SERS method developed in this study.

### 3.3. Simultaneous Detection of MC and 4-MMC in Water

**Figure 3:**
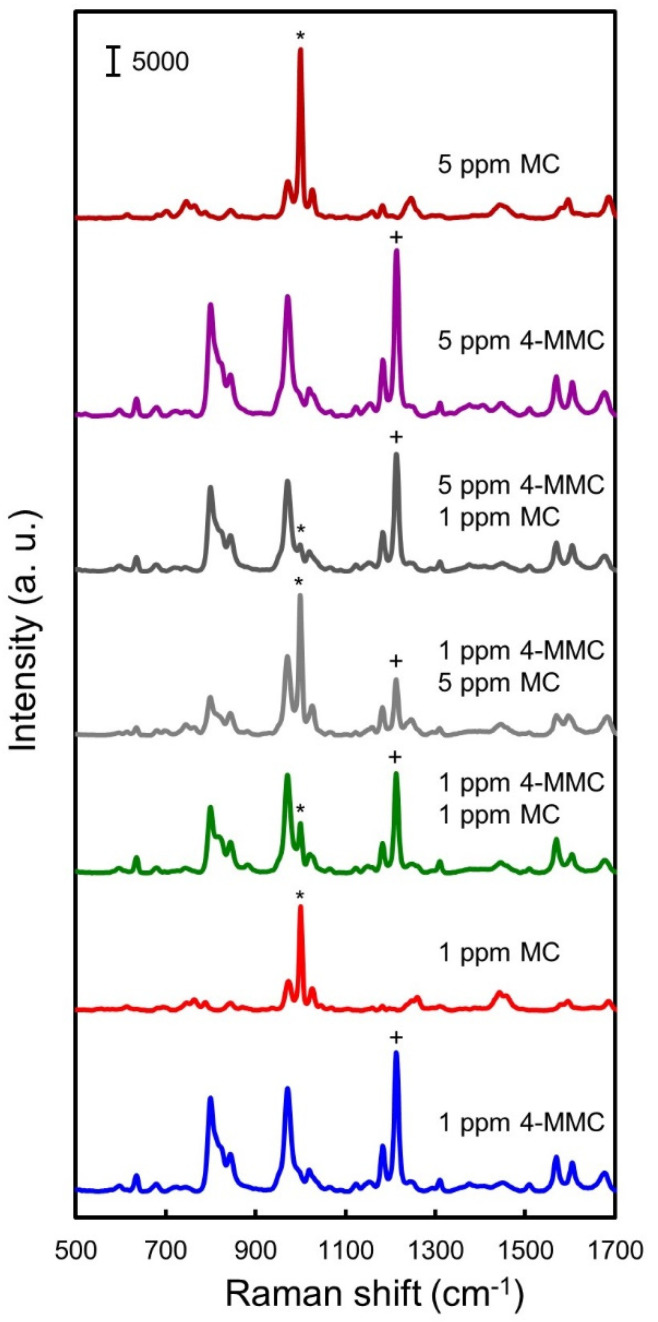
SERS detection of MC, 4-MMC, and mixtures of MC and 4-MMC in water. SERS spectra of 1 ppm MC, 5 ppm MC, 1 ppm 4-MMC, 5 ppm 4-MMC, 5 ppm 4-MMC plus 1 ppm MC, 1 ppm 4-MMC plus 5 ppm MC, and 1 ppm 4-MMC plus 1 ppm MC. The characteristic Raman peak of MC at 1000 cm^−1^ and the characteristic Raman peak of 4-MMC at 1212 cm^−1^ are marked with “*” and “+”, respectively.

**Figure 4:**
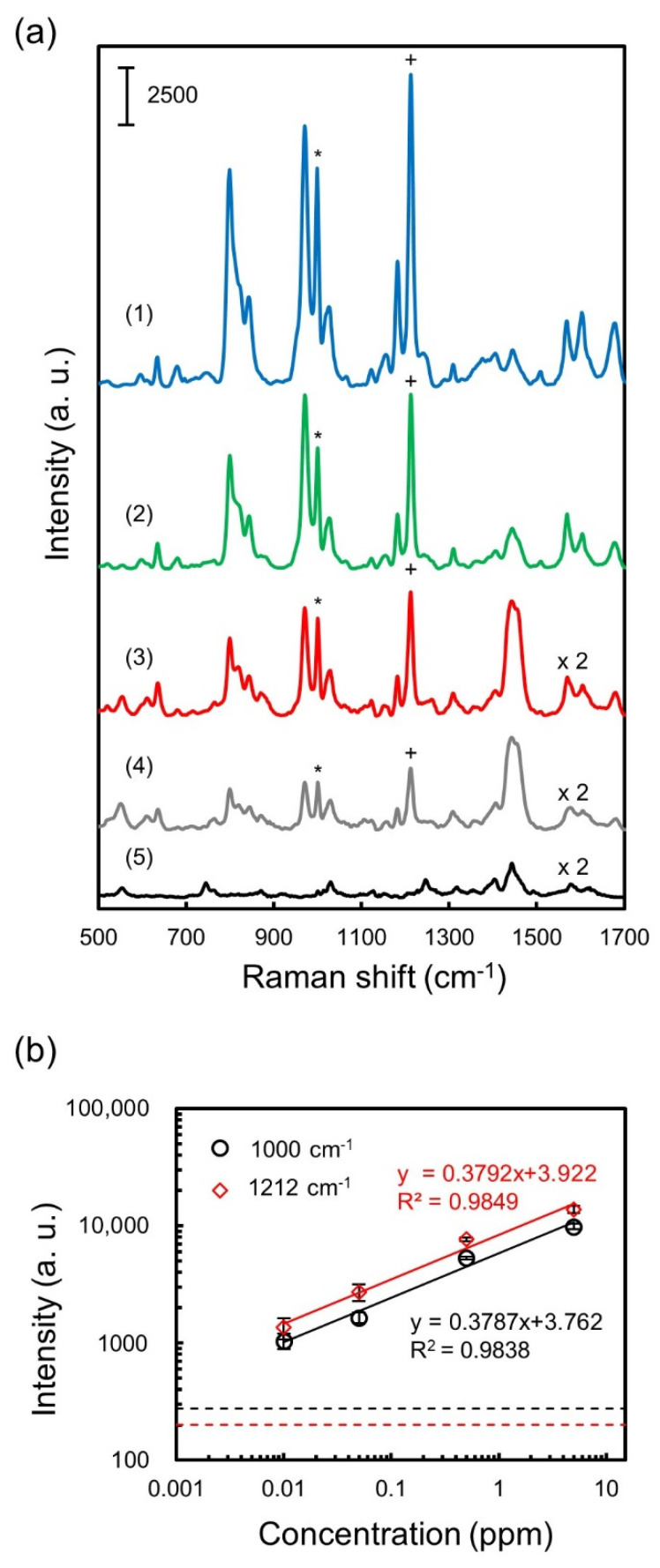
Simultaneous SERS detection of MC and 4-MMC in water. (**a**) SERS spectra of mixtures of MC and 4-MMC. The concentration of both MC and 4-MMC was 5, 0.5, 0.05, 0.01, and 0 ppm for the SERS spectrum (1), (2), (3), (4), and (5), respectively. The characteristic Raman peak of MC at 1000 cm^−1^ and the characteristic Raman peak of 4-MMC at 1212 cm^−1^ are marked with “*” and “+”, respectively. (**b**) A plot of the intensity of the 1000 cm^−1^ peak versus MC concentration and the intensity of the 1212 cm^−1^ peak versus 4-MMC concentration. Error bars show standard deviations with n = 30. The straight lines are the linear regression lines, and the black and red dotted lines indicate mean plus 3σ of the 1000 and the 1212 cm^−1^ peak, respectively, of the blank sample.

In addition to demonstrating the SERS detection of each of the two kinds of drugs, we also tested the simultaneous detection of both MC and 4-MMC. Compared with immunoassay methods, simultaneous detection of multiple kinds of drugs is an advantage for SERS-based method because it does not require multiple kinds of antibodies for detection. Multiple drugs can be detected simultaneously using SERS as long as their characteristic Raman peaks do not fully overlap with each other. To test the simultaneous detection of MC and 4-MMC, we measured the SERS spectra of three kinds of drug mixtures, including 5 ppm 4-MMC plus 1 ppm MC, 1 ppm 4-MMC plus 5 ppm MC, and 1 ppm 4-MMC plus 1 ppm MC. We also measured 1 ppm MC, 5 ppm MC, 1 ppm 4-MMC, and 5 ppm 4-MMC for comparison. The results, as shown in Figure 3, revealed that the SERS spectra of the mixtures had the characteristic Raman peaks of both drugs. It should be noted that, because the adsorption of one kind of molecule to the AgNPs could interfere with the adsorption of other molecules, the SERS spectra measured for the mixtures of MC and 4-MMC showed weaker characteristic Raman peaks than the SERS spectra of individual drugs of the same concentration. In addition, our results showed that the 4-MMC molecule appeared to have stronger adsorption to the AgNPs than the MC molecule because the intensities of its characteristic Raman peaks decreased less during the simultaneous detection of 4-MMC and MC. To determine the limits of detection during simultaneous detection, we performed SERS measurements of 4-MMC and MC of various concentrations. Figure 4a shows the SERS spectra of the mixtures of MC and 4-MMC, and Figure 4b shows the relationships between the concentration of each drug in the mixture and the intensities of the characteristic Raman peaks. The results showed that, although the intensities of the characteristic Raman peaks of MC and 4-MMC were weaker when the two drugs were detected simultaneously, we could still detect both drugs at a concentration as low as 0.01 ppm in the mixture.

### 3.4. Urine Sample Pretreatment

**Figure 5:**
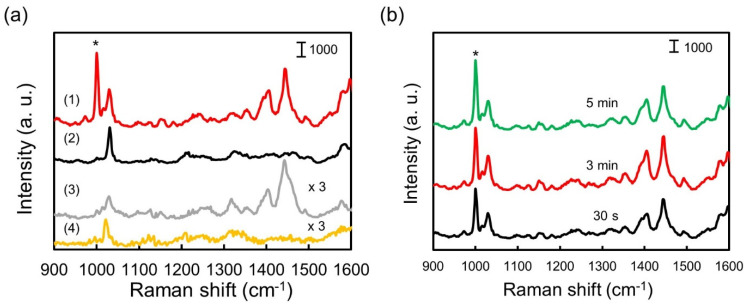
Effects of the sample pretreatment process on detection of drugs in urine. (**a**) SERS spectra of 0.1 ppm MC in urine measured (1) after and (2) before sample pretreatment and SERS spectra of blank urine samples measured (3) after and (4) before sample pretreatment. (**b**) SERS detection of 0.1 ppm MC in urine with different extraction time. The characteristic Raman peak of MC at 1000 cm^−1^ is marked with “*”.

While we already demonstrated the sensitive detection of MC and 4-MMC standard solutions, to detect drugs in human urine samples, we had to use a suitable sample pretreatment method before the SERS measurements to reduce the interferences from other components in urine. As shown in Figure 5a, when using our SERS method to measure urine samples containing 0.1 ppm MC, which was already 10 times higher than the lowest detected concentration shown in Figure 2a, we could not detect the 1000 cm^−1^ Raman peak of MC unless the urine samples were processed using the sample pretreatment method illustrated in Scheme 1. The results confirmed that using the sample pretreatment process to extract and concentrate drug molecules was essential for achieving sensitive detection of drugs in urine samples. Figure 5b shows that, when the time duration for mixing hexane and the urine samples containing 0.1 ppm MC increased from 30 s to 3 min and 5 min, the intensity of the characteristic Raman peak at 1000 cm^−1^ did not significantly increase with the mixing time. Therefore, to reduce the time of the sample pretreatment as much as possible, we only mixed hexane with urine samples for 30 s for latter experiments.

### 3.5. Simultaneous Detection of MC and 4-MMC in Urine

**Figure 6:**
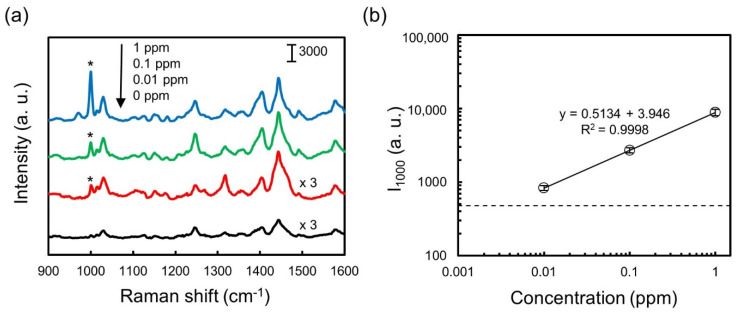
SERS detection of MC in water after sample pretreatment. (**a**) SERS spectra of MC in water measured after sample pretreatment. The characteristic Raman peak of MC at 1000 cm^−1^ is marked with “*”. (**b**) A plot of the intensity of the 1000 cm^−1^ peak versus MC concentration. Error bars show standard deviations with *n* = 30. The straight line is the linear regression line, and the dotted line indicates mean plus 3σ of the 1000 cm^−1^ peak of the blank sample.

**Figure 7:**
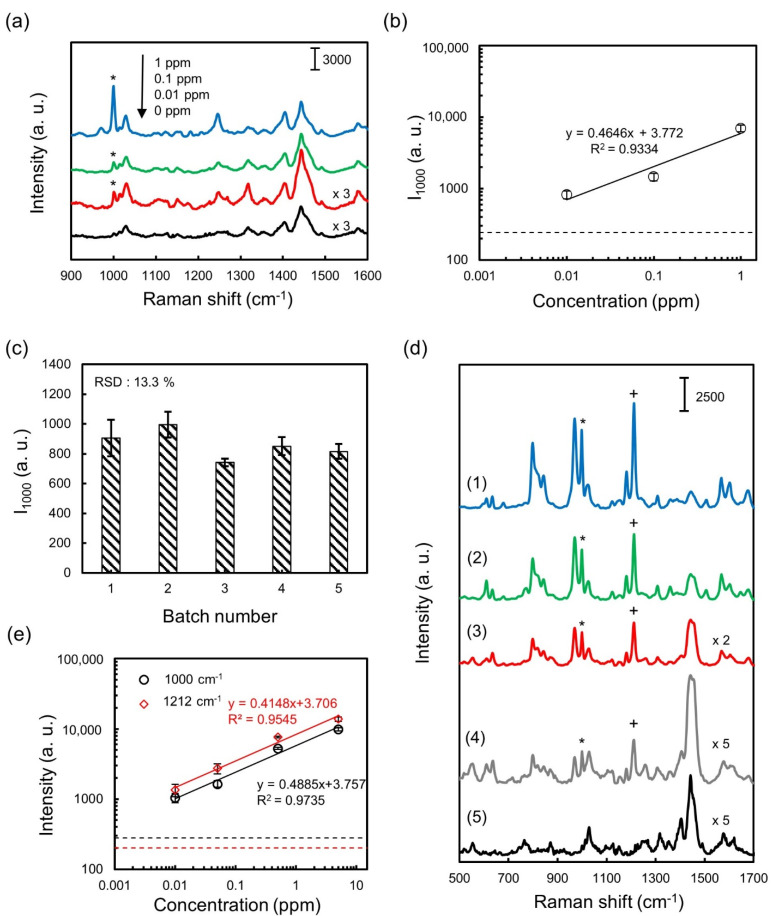
SERS detection of MC and 4-MMC in urine. (**a**) SERS spectra of MC in urine measured after sample pretreatment. The characteristic Raman peak of MC at 1000 cm^−1^ is marked with “*”. (**b**) A plot of the intensity of the 1000 cm^−1^ peak versus MC concentration. Error bars show standard deviations with n = 30. The straight line is the linear regression line, and the dotted line indicates mean plus 3σ of the 1000 cm^−1^ peak of the blank sample. (**c**) The intensities of the 1000 cm^−1^ peak measured from 5 urine samples containing 0.01 ppm MC. Error bars show standard deviations with n = 10. (**d**) SERS spectra of urine samples containing both MC and 4-MMC measured after sample pretreatment. The concentration of both MC and 4-MMC was 5, 0.5, 0.05, 0.01, and 0 ppm for the SERS spectrum (1), (2), (3), (4), and (5), respectively. The characteristic Raman peak of MC at 1000 cm^−1^ and the characteristic Raman peak of 4-MMC at 1212 cm^−1^ are marked with “*” and “+”, respectively. (**e**) A plot of the intensity of the t 1000 cm^−1^ peak versus MC concentration and the intensity of the 1212 cm^−1^ peak versus 4-MMC concentration. Error bars show standard deviations with n = 10. The straight lines are the linear regression lines, and the black and red dotted lines indicate mean plus 3σ of the 1000 and the 1212 cm^−1^ peak, respectively, of the blank sample.

We further tested the sample pretreatment method with standard solutions and urine samples containing drugs of different concentrations. We first prepared 1, 0.1, and 0.01 ppm MC standard solutions and processed them using the sample pretreatment method. Although these samples were not urine samples, we pretreated them before measuring their SERS spectra to understand how the detection results would be affected by the sample pretreatment process. As shown in Figure 6a, the results of the SERS measurements revealed that 0.01 ppm MC could still be detected after the sample pretreatment. Figure 6b shows that the intensity of the 1000 cm^−1^ peak increased with the concentration of MC. Then, to test the detection of drugs in urine samples, we prepared urine samples containing 1, 0.1, and 0.01 ppm MC and then pretreated the samples using the same method before the SERS measurements. Figure 7a,b show that we could successfully detect MC in urine samples containing 0.01 ppm MC or more. The relation between the intensity of the 1000 cm^−1^ peak and MC concentration shown in Figure 7b was slightly different from that shown in Figure 6b because they were measured with urine samples and with water samples, respectively. To understand the reproducibility of the SERS measurements, we prepared five urine samples containing 0.01 ppm MC and then performed 10 repeated measurements for each sample. Figure 7c shows the intensities of the 1000 cm^−1^ peak in the SERS spectra (Supplementary material Figure S4) of the five urine samples and the relative standard deviation (RSD) of the results, which was 13.3%. Finally, we prepared urine samples containing both MC and 4-MMC to test the simultaneous extraction and detection of both drugs in urine. Figure 7d shows that the SERS spectra of the extracted solutions presented the characteristic Raman peaks of both drugs, including the 1000 cm^−1^ peak of MC and the 1212 cm^−1^ peak of 4-MMC. The concentrations of MC and 4-MMC in the tested urine samples were in the range of 0.01–5 ppm. In contrast, the SERS spectra of the blank sample, which was the urine sample without drug additives, did not present the characteristic Raman peaks of the drugs. Figure 7e shows that the intensity of the 1000 and the 1212 cm^−1^ peak increased with the concentration of MC and 4-MMC. 

Our results show that we could simultaneously detect MC and 4-MMC in urine at a concentration as low as 0.01 ppm. In comparison, Muhamadali et al. demonstrated the detection of 4-MMC in water and in urine with limits of detection of ~800 ppm by mixing AgNPs with sample solutions [35]. No sample pretreatment method was used in their study. Han et al. reported SERS detection of 10 ppm MC and two other kinds of drugs in urine using self-assembled gold nanorod arrays, but they did not determine the limit of detection for MC [25]. Lee et al. showed that their polymer-stabilized Ag nanoparticle films could be used as SERS substrates to detect 5.7 μg of MC using a swabbing method [34]. In addition to the SERS-based methods, an electrochemical method was used to detect 4-MMC with a limit of detection of 11.8 ppm [46]. In comparison with these results, the SERS method developed in this study was one of the most sensitive rapid screening methods reported in the literature for the detection of MC and 4-MMC in urine.

## 4. Conclusions

In conclusion, we have successfully developed a rapid, low-cost, and sensitive method for the detection of MC and 4-MMC in urine. To reduce the interferences from other components in urine on the detection results, we used a simple sample pretreatment process that could extract and concentrate drugs from urine within 20 min before SERS measurements. To achieve the sensitive detection of drugs, we optimized the kind and concentration of the alkali halide salt used to quickly form Ag nanoclusters with strong SERS effects. Our results showed that, by using NaBr as the aggregating agents for AgNPs, we could simultaneously detect both MC and 4-MMC in urine at a concentration as low as 0.01 ppm after sample pretreatment. The whole measurement process could be finished within 1 min because we measured the SERS spectra immediately after we added the AgNPs and NaBr to the extracted sample solution without incubation. We believe that the SERS-based detection method and the sample pretreatment process developed in this study have a great potential to be used for rapid screening of illegal drugs in urine.

## Data Availability

The data presented in this study are available on request from the corresponding author.

## Supplementary Materials

The following are available online at https://www.mdpi.com/article/10.3390/nano11071789/s1, Figure S1: Extinction spectra and TEM image of the AgNPs, Figure S2: Hydrodynamic diameter of the AgNPs determined using dynamic light scattering (DLS), Figure S3: Time-lapse observation of SERS spectra after the addition of NaBr. Figure S4: SERS spectra of 5 urine samples containing 0.01 ppm MC.
