# Supplementary material for: Rapid Formation of Nanoclusters for Detection of Drugs in Urine Using Surface-Enhanced Raman Spectroscopy
Source: Nanomaterials (Basel). 2021 Jul 9;11(7):1789. doi: 10.3390/nano11071789 (PMC8308440; doi:10.3390/nano11071789)
Supplement: Supplementary file 1 [file nanomaterials-11-01789-s001.zip › nanomaterials-1274065-supplementary.pdf]

Supplementary Materials

Rapid Formation of Nanoclusters for Detection of Drugs in Urine Using Surface-Enhanced Raman Spectroscopy

Yun-Chu Chen[1], Shun-Wen Hong[1], Hong-Shin Wu[1], Yuh-Lin Wang[2], Yih-Fan Chen[1], *

[1] Institute of Biophotonics, National Yang Ming Chiao Tung University, Taipei 112, Taiwan
[2] Institute of Atomic and Molecular Sciences, Academia Sinica, Taipei 106, Taiwan
* Correspondence: chenyf@nycu,edu.tw

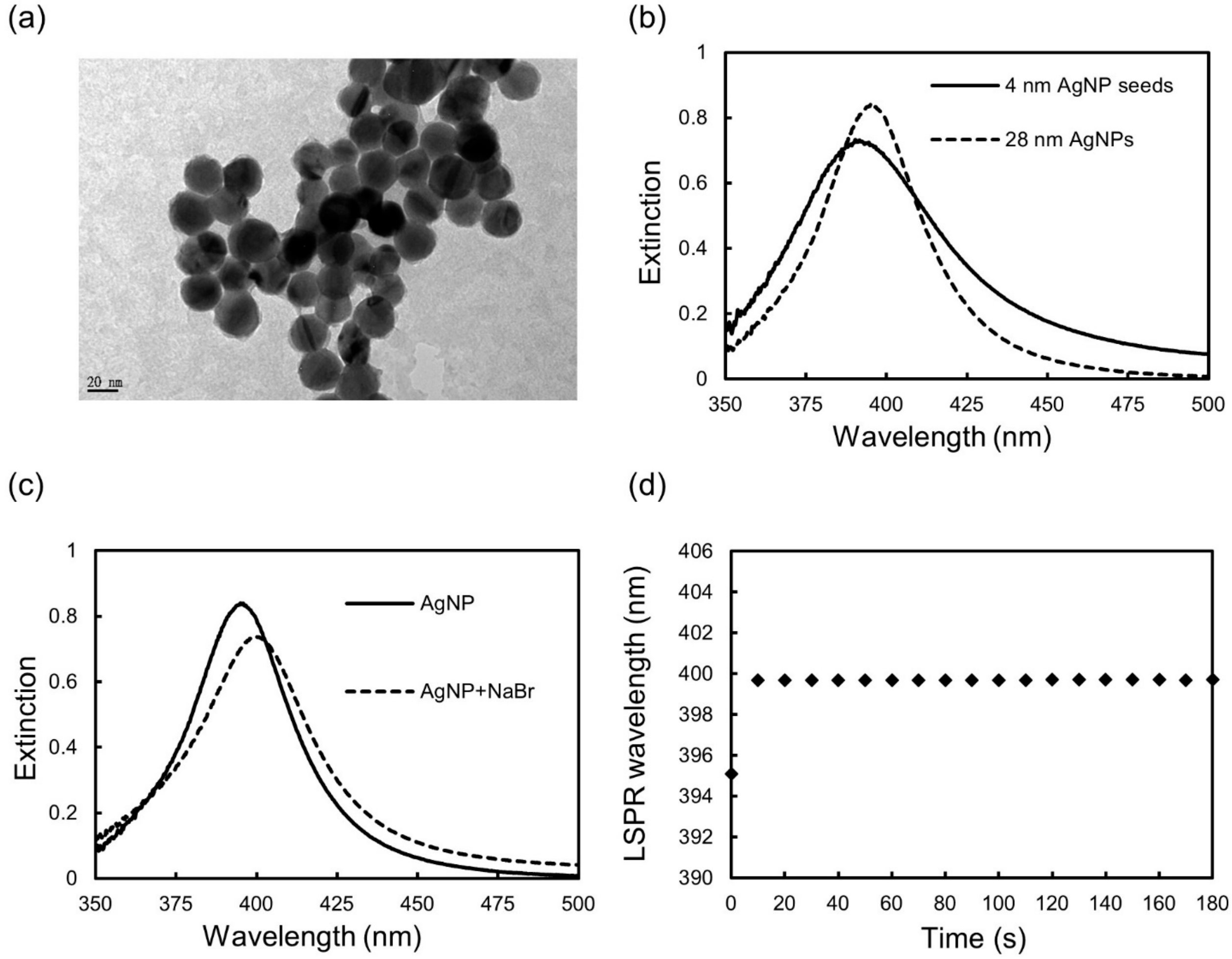

Figure S1. TEM image and extinction spectra of the AgNPs. (a) Representative TEM image of the 28 nm AgNPs. (b) Extinction spectra of the 4 nm AgNP seeds and the 28-nm AgNPs. The LSPR peaks of the AgNPs were 391.9 nm and 395.1 nm for the 4 nm AgNP seeds and the 28 nm AgNPs, respectively. (c) Extinction spectra of the AgNPs before and after mixed with 12 mM NaBr. (d) LSPR peaks of the AgNPs before and after mixed with 12 mM NaBr. NaBr was added to the AgNP solution at t = 0 s. The LSPR peak of the AgNPs changed from 395.1 m to 399.7 nm after the addition of NaBr.

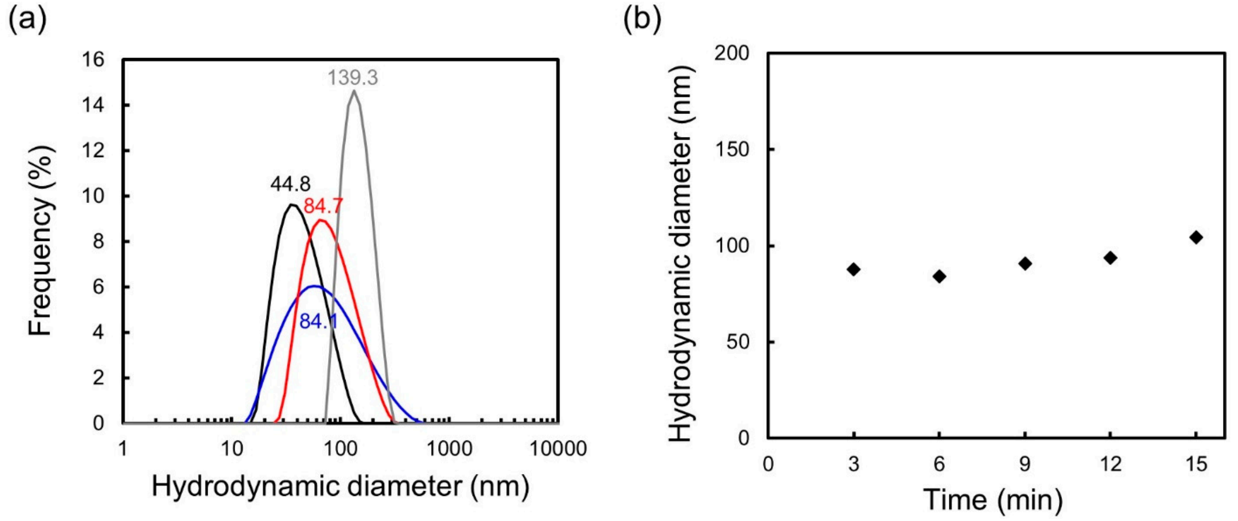

Figure S2. Hydrodynamic diameter of the AgNPs determined by dynamic light scattering (DLS). (a) Hydrodynamic diameters of the AgNPs without NaBr (black) and the AgNPs mixed with 6 mM NaBr (blue), 12 mM NaBr (red), and 24 mM NaBr (gray). (b) Time-lapse variation of the hydrodynamic diameter of the AgNPs mixed with 12 mM NaBr.

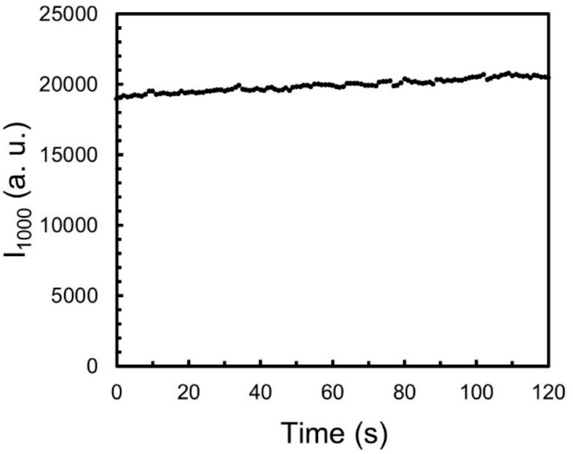

Figure S3. Time-lapse observation of SERS spectra after the addition of NaBr. A plot of the intensity of the characteristic Raman peak of MC at 1000 cm^{-1} versus time after adding 12 mM NaBr to a mixture of 1 ppm MC and AgNPs.

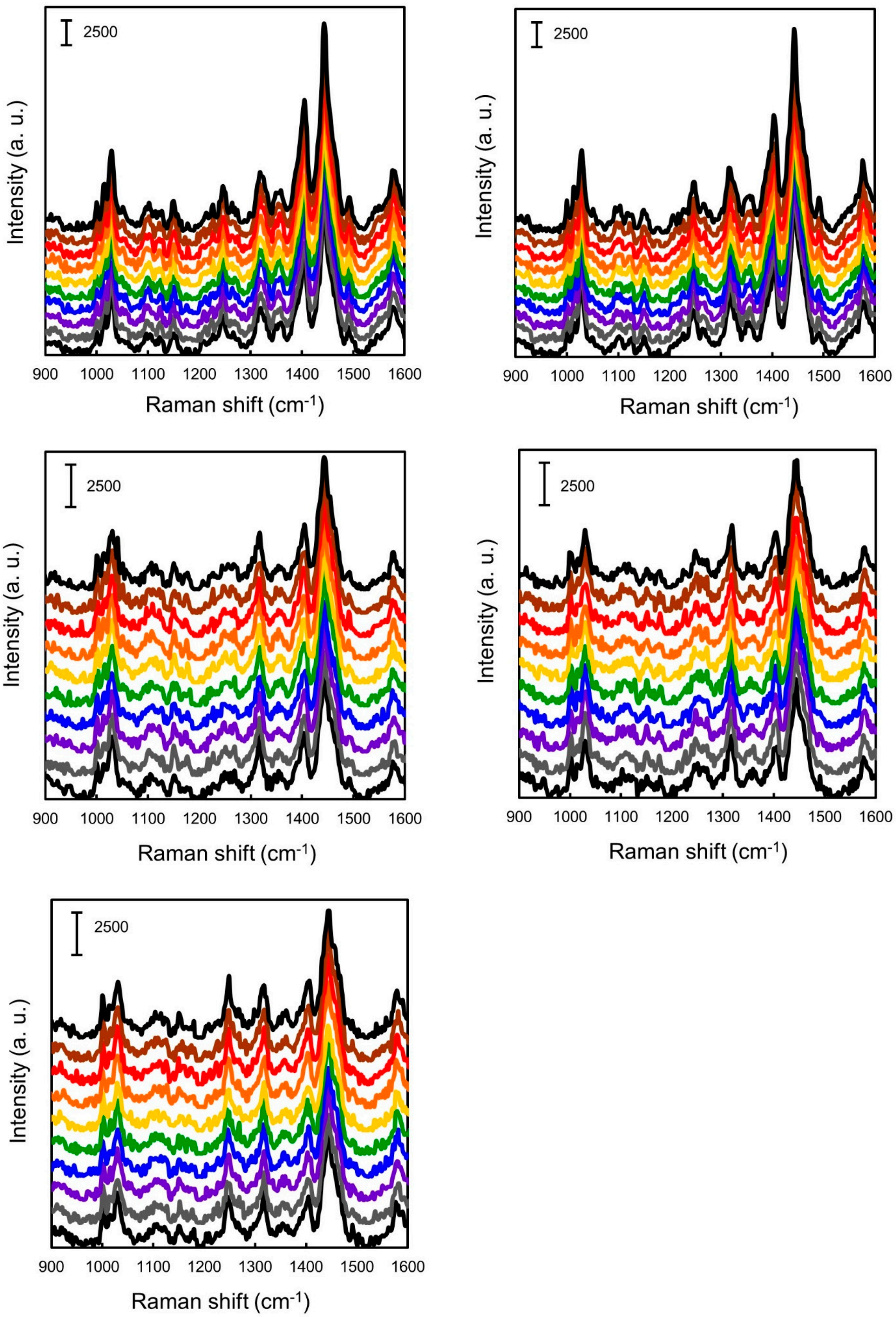

Figure S4. SERS spectra of 5 urine samples containing 0.01 ppm MC. The intensities of the characteristic Raman peak and the RSD value of the results are shown in Figure 7c.
